# Studies on the Anticonvulsant Activity and Influence on GABA-ergic Neurotransmission of 1,2,4-Triazole-3-thione-Based Compounds

**DOI:** 10.3390/molecules190811279

**Published:** 2014-07-31

**Authors:** Tomasz Plech, Barbara Kaproń, Jarogniew J. Łuszczki, Monika Wujec, Agata Paneth, Agata Siwek, Marcin Kołaczkowski, Maria Żołnierek, Gabriel Nowak

**Affiliations:** 1 Department of Organic Chemistry, Medical University of Lublin, Chodźki 4a, Lublin 20-093, Poland; E-Mails: baska_k@o2.pl (B.K.); monika.wujec@umlub.pl (M.W.); agata.siwek@umlub.pl (A.P.); 2 Department of Pathophysiology, Medical University of Lublin, Jaczewskiego 8, Lublin 20-090; Poland; E-Mail: jarogniew.luszczki@gmail.com; 3 Isobolographic Analysis Laboratory, Institute of Rural Health, Jaczewskiego 2, Lublin 20-950, Poland; 4 Department of Pharmacobiology, Jagiellonian University Medical College, Medyczna 9, Kraków 30-688, Poland; E-Mails: asiwku@wp.pl (A.S.); mfzolnie@cyf-kr.edu.pl (M.Ż.); nowak@if-pan.krakow.pl (G.N.); 5 Department of Pharmaceutical Chemistry, Jagiellonian University Medical College, Medyczna 9, Kraków 30-688, Poland; E-Mail: mkolacz@tlen.pl

**Keywords:** 30-688 *s*-triazoles, Mannich bases, maximal electroshock-induced seizure (MES) test, blood-brain barrier (BBB), radioligand binding assay

## Abstract

The anticonvulsant activity of several 1,2,4-triazole-3-thione derivatives on mouse maximal electroshock-induced seizures was tested in this study. Characteristic features of all active compounds were rapid onset of action and long lasting effect. Structure-activity observations showed that the probability of obtaining compounds exerting anticonvulsant activity was much higher when at least one of the phenyl rings attached to 1,2,4-triazole nucleus had a substituent at the *para* position. The obtained results, moreover, permit us to conclude that despite the structural similarity of loreclezole (second-generation anticonvulsant drug) and the titled compounds, their anticonvulsant activity is achieved via completely different molecular mechanisms.

## 1. Introduction

Epilepsy is a chronic disease of the central nervous system caused by paroxysmal, recurrent disturbances of the bioelectrical activity of the brain. It is estimated that approximately 50 million people worldwide have epilepsy [1]. An important point is the fact that epilepsy affects young children and adolescents more than any other age group [2]. As epileptic seizures are the result of an imbalance between neurotransmitters in the brain, the mechanism of action of some antiepileptic drugs consists in modulating the activity of GABA-ergic and glutamatergic systems [3]. Moreover, a large group of antiepileptic drugs is constituted by substances influencing the activity of voltage-dependent sodium channels [4,5,6,7]. The same drug is often capable of influencing different molecular targets (receptors, enzymes, *etc.*), which makes it difficult to define these elements of the drug’s structure, which determine its pharmacological activity to the largest extent.

However, extensive theoretical studies aimed at defining the relationship between molecular structure and bioactivity of the antiepileptic drugs available on the market have shown that (i) spatially remote hydrophobic domains (usually phenyl rings); (ii) the hydrogen bonding domain; and (iii) the electron-donating fragment constitute the elements that are necessary for good anticonvulsant activity [8,9,10]. On the other hand, Wingrove and co-workers put forward a hypothesis that the activity of loreclezole (second-generation antiepileptic drug) is dependent on the interaction between the triazole moiety and the amide group of asparagine (Asn-289), which is located on the β_2_ subunit of the GABA_A_ receptor [11]. Our pilot studies moreover showed that 1,2,4-triazole derivatives administered systemically in completely non-toxic doses, significantly elevated the threshold for electroconvulsions and intensified the anticonvulsant activity of classic antiepileptic drugs [12,13]. Many other compounds bearing a 1,2,4-triazole moiety were also found to possess anticonvulsant properties in various animal models of epilepsy [14,15,16,17]. These observations inspired us to: (i) search for triazole-based compounds endowed with anticonvulsant activity; and (ii) to check whether the obtained derivatives may act via a mechanism analogous to that of loreclezole, based on the allosteric modulation of GABA_A_ receptors. The results thus obtained also served as the grounds of theoretical considerations on structural requirements conditioning anticonvulsant activity of 1,2,4-triazole-3-thione derivatives.

## 2. Results and Discussion

### 2.1. Chemistry

**Scheme 1:**
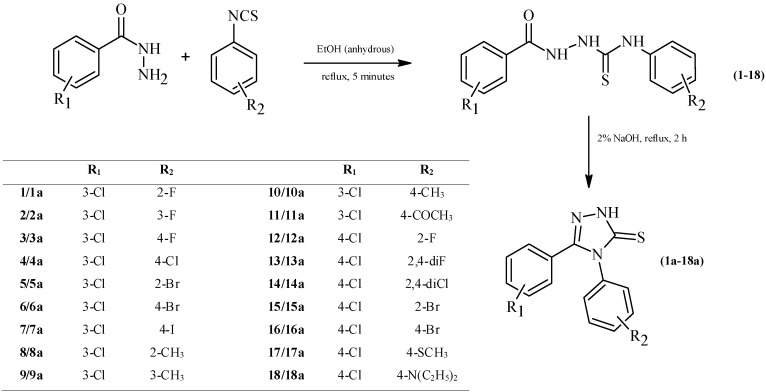
Synthetic pathway for the synthesis of 1,2,4-triazole-3-thione derivatives.

**Scheme 2:**
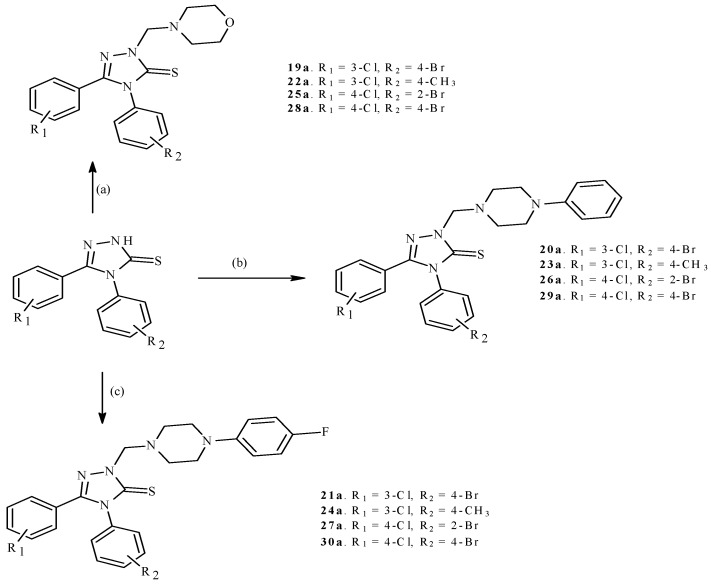
Synthetic route to 1,2,4-triazole based Mannich bases (**19a**–**30a**). Reagents and conditions: (**a**) morpholine, HCHO, EtOH, 1 h, r.t.; (**b**) 1-phenylpiperazine, HCHO, EtOH, 1 h, r.t.; (**c**) 1-(4-fluorophenyl)piperazine, HCHO, EtOH, 1 h, r.t.

The synthetic pathway of 1,2,4-triazole-3-thione based compounds is presented in Scheme 1 and Scheme 2. The substrates for the synthesis were the commercially available hydrazides, 3-chlorobenzhydrazide and 4-chlorobenzhydrazide. Reaction between the above-mentioned hydrazides and appropriate aryl isothiocyanates yielded 1,4-disubstituted thiosemicarbazide derivatives (**1**–**18**). The use of a highly efficient and quick method of synthesis [18] in anhydrous ethanol allowed us to obtain thiosemicarbazides (**1**–**18**) with an efficiency exceeding 85% in most cases. Subsequently, compounds (**1**–**18**) were dissolved in 2% NaOH solution and heated under reflux for 2 h, which resulted in obtaining 4,5-diaryl-1,2,4-triazole-3-thiones (**1a**–**18a**). Finally, the compounds (**19a**–**30a**) were synthesized using the so-called Mannich reaction. Its mechanism has been extensively described by Almajan and co-workers [19]. The structures of the obtained compounds were confirmed on the basis of spectroscopic data and the results of elemental analyses.

### 2.2. Pharmacology

#### 2.2.1. Anticonvulsant Activity

Compounds (**1a**–**30a**) were evaluated *in vivo* using the mouse maximal electroshock-induced seizure model-an experimental model of human generalized tonic-clonic seizures. Procedures involving animals and their care were conducted in accordance with current European Community and Polish legislation on animal experimentation. Additionally, all efforts were made to minimize animal suffering and to use only the number of animals necessary to produce reliable scientific data.

In the initial stage of the study, groups of eight mice were supplied with the compounds (administered *intraperitoneally*) in a dose of 300 mg/kg and then exposed to electrical impulse at fixed intervals (15, 30, 60, 120 min). The anticonvulsant effects of the compounds were considered important if at least 50% of the animals tested were protected against electroshock-induced seizures (Table 1). In the cases of compounds **3a** and **14a**, administered in a dose of 300 mg/kg, it was impossible to establish their protective effect in the maximal electroshock-induced seizure (MES) test, as the dose resulted in the occurrence of acute adverse effects. Once the doses of both compounds were decreased to lower concentrations, which did not induce neurotoxic effects, only Compound **3a** retained its anticonvulsant activity. Nine of the 30 tested compounds (**1a**–**30a**) exhibited various levels of anticonvulsant activity. All active derivatives (**3a**, **4a**, **6a**, **10a**, **12a**, **13a**, **15a**, **16a**, **22a**) were characterized by a quick onset of action. A significant level of protection against maximal-electroshock induced seizures was observed as early as 15 min after their parenteral administration. From a preclinical point of view, the long-lasting anticonvulsant activity is also noteworthy. Apparently, the highest ability to remain in the central nervous system was presented by **13a** and **16a**, whose time to peak of the anticonvulsant action was observed at 120 min after their systemic administration.

In the second stage of the pharmacological research median effective doses (ED_50_), median toxic doses (TD_50_), and protective index (PI) values for compounds **3a**, **4a**, **6a**, **10a**, **12a**, **13a**, **15a**, **16a**, and **22a** were determined (Table 2). The most potent activity (approximately five and a half times higher than that of valproate) was exhibited by 5-(3-chlorophenyl)-4-(4-fluorophenyl)-2,4-dihydro-3*H*-1,2,4-triazole-3-thione (**3a**) whose ED_50_ equaled 35.2 mg/kg. Time-course and dose-response relationship of action of **3a** revealed that the time to peak of anticonvulsant action was observed at 15 min after its *i.p.* administration. The level of toxicity (TD_50_) of the compound, ranging from 136.7 to 201.0 mg/kg makes long-term use of this derivative impossible. However, due to the fast onset of action, low ED_50_ value, as well as the favorable protection index value (PI = 3.9), Compound **3a** should undergo further investigation for its potential use in interrupting *status epilepticus*. Among the remaining derivatives, only compounds **6a** and **22a** exhibited activity slightly weaker than that of valproate. The former compound, despite its less potent anticonvulsant activity, significantly (*i.e*., by 70%) elevated the threshold for electroconvulsions and intensified the anticonvulsant action of carbamazepine, phenobarbital, and valproate [12]. A very beneficial activity profile, expressed by low variability in the ED_50_ values at different pretreatment times (15, 30, 60, 120 min), was observed in the cases of 2-fluorophenyl (**12a**) and 2,4-difluorophenyl (**13a**) derivatives. The ED_50_ values for these derivatives were 186.4–217.8 mg/kg and 80–108.9 mg/kg, respectively. It is also noteworthy that 5-(4-chlorophenyl)-4-(2-fluorophenyl)-2,4-dihydro-3*H*-1,2,4-triazole-3-thione (**12a**) was the best tolerated compound among all tested 1,2,4-triazole derivatives. Its median toxic doses (TD_50_) ranged from 418.5 to 534.3 mg/kg. In most cases, the toxicological profile of the discussed compounds was similar to that of valproate (PI = 1.7–2.1). A more beneficial value of the protection index (PI = 4.5–5.9) was observed for 5-(3-chlorophenyl)-4-(4-methylphenyl)-2,4-dihydro-3*H*-1,2,4‑triazole-3-thione (**10a**). Additionally, our previous studies demonstrated pharmacologically beneficial interactions of **10a** with valproate that were pharmacokinetic in nature [13]. Sub-protective doses (*i.e*., the doses that *per se* did not affect the seizure threshold) of compound **10a** intensified the anticonvulsant action of valproate as the total brain concentration of this drug was elevated by 52%.

**Table 1:** Time-course of anticonvulsant effects of compounds (**1a**–**30a**) against maximal electroshock-induced seizure (MES)-induced seizures in mice. Data is presented as the number of animals protected against maximal electroshock (MES)-induced seizures out of eight animals per group. The MES test was performed at various pretreatment times (15, 30, 60, 120 min) after systemic administration of the investigated compounds in a fixed dose of 300 mg/kg. *p*-number of animals protected against MES-induced seizures; t-number of animals tested. “*” - Substance administered at a dose of 300 mg/kg produced acute neurotoxic effects in mice and it was impossible to determine its anticonvulsant action in the mouse MES model.

Compounds	Pretreatment Time (min)			
	15	30	60	120
	p/t (%)	p/t (%)	p/t (%)	p/t (%)
**1a**	0/8 (0)	0/8 (0)	1/8 (12.5)	1/8 (12.5)
**2a**	0/8 (0)	0/8 (0)	0/8 (0)	0/8 (0)
**3a**	*	*	*	*
**4a**	8/8 (100)	8/8 (100)	7/8 (87.5)	7/8 (87.5)
**5a**	0/8 (0)	0/8 (0)	1/8 (12.5)	1/8 (12.5)
**6a**	4/8 (50)	6/8 (75)	6/8 (75)	3/8 (37.5)
**7a**	0/8 (0)	0/8 (0)	0/8 (0)	0/8 (0)
**8a**	0/8 (0)	0/8 (0)	0/8 (0)	0/8 (0)
**9a**	0/8 (0)	0/8 (0)	0/8 (0)	1/8 (12.5)
**10a**	8/8 (100)	8/8 (100)	8/8 (100)	6/8 (75)
**11a**	1/8 (12.5)	1/8 (12.5)	1/8 (12.5)	0/8 (0)
**12a**	6/8 (75)	6/8 (75)	7/8 (87.5)	7/8 (87.5)
**13a**	8/8 (100)	8/8 (100)	8/8 (100)	8/8 (100)
**14a**	*	*	*	*
**15a**	6/8 (75)	8/8 (100)	8/8 (100)	7/8 (87.5)
**16a**	5/8 (62.5)	7/8 (87.5)	8/8 (100)	8/8 (100)
**17a**	0/8 (0)	0/8 (0)	1/8 (12.5)	0/8 (0)
**18a**	0/8 (0)	0/8 (0)	1/8 (12.5)	0/8 (0)
**19a**	0/8 (0)	0/8 (0)	0/8 (0)	0/8 (0)
**20a**	0/8 (0)	0/8 (0)	0/8 (0)	0/8 (0)
**21a**	0/8 (0)	0/8 (0)	0/8 (0)	0/8 (0)
**22a**	4/8 (50)	5/8 (62.5)	3/8 (37.5)	2/8 (25)
**23a**	0/8 (0)	0/8 (0)	0/8 (0)	0/8 (0)
**24a**	0/8 (0)	0/8 (0)	0/8 (0)	0/8 (0)
**25a**	0/8 (0)	0/8 (0)	0/8 (0)	0/8 (0)
**26a**	0/8 (0)	0/8 (0)	0/8 (0)	0/8 (0)
**27a**	0/8 (0)	0/8 (0)	0/8 (0)	0/8 (0)
**28a**	0/8 (0)	0/8 (0)	0/8 (0)	0/8 (0)
**29a**	0/8 (0)	0/8 (0)	0/8 (0)	0/8 (0)
**30a**	0/8 (0)	0/8 (0)	0/8 (0)	0/8 (0)

**Table 2:** Time-course and dose-response effects of the active compounds in the MES and chimney tests in mice. Results are presented as median effective doses (ED_50_ ± S.E.) and median toxic doses (TD_50_ ± S.E.) of the examined compounds. All compounds were administered *i.p.*, at four various pretreatment times (15, 30, 60, 120 min.) prior to the appropriate pharmacological test (MES or chimney test). n - the total number of animals used at those doses whose anticonvulsant/toxic effects ranged between 4 and 6 probits.

Compounds	PretreatmentTime (min)	ED_50_ ± S.E.(mg/kg)	n	TD_50_ ± S.E.(mg/kg)	n	PI (TD_50_/ED_50_)
**3a**	15	35.2 ± 5.3	8	136.7 ± 19.9	24	3.9
	30	93.6 ± 9.4	16	148.8 ± 19.9	32	1.6
	60	>200	-	171.3 ± 21.4	24	-
	120	>200	-	201.0 ± 20.3	32	-
**4a**	15	90.1 ± 15.7	16	354.9 ± 21.5	24	3.9
	30	135.0 ± 19.8	8	365.3 ± 24.0	16	2.7
	60	171.3 ± 21.4	24	361.0 ± 20.2	32	2.1
	120	183.8 ± 28.0	24	375.8 ± 26.1	32	2.0
**6a**	15	297.5 ± 21.1	24	372.5 ± 22.6	16	1.2
	30	217.8 ± 21.7	16	383.7 ± 18.7	16	1.8
	60	208.9 ± 27.3	32	389.3 ± 19.1	16	1.9
	120	335.7 ± 14.0	32	424.7 ± 21.4	32	1.3
**10a**	15	57.0 ± 9.4	16	338.1 ± 12.0	24	5.9
	30	74.5 ± 8.1	16	338.1 ± 14.7	16	4.5
	60	187.1 ± 18.8	16	333.4 ± 18.6	16	1.8
	120	281.4 ± 13.6	32	395.1 ± 25.2	24	1.4
**12a**	15	207.5 ± 25.3	32	534.3 ± 25.9	40	2.6
	30	186.4 ± 24.6	32	496.8 ± 31.7	32	2.7
	60	217.8 ± 21.7	16	418.5 ± 34.3	32	1.9
	120	208.9 ± 27.3	32	483.8 ± 25.9	40	2.3
**13a**	15	104.0 ± 15.8	8	195.7 ± 21.8	8	1.9
	30	100.0 ± 30.1	8	191.2 ± 21.1	8	1.9
	60	108.9 ± 11.9	16	176.1 ± 17.2	16	1.6
	120	80.0 ± 10.9	24	217.8 ± 21.7	16	2.7
**15a**	15	182.7 ± 25.1	24	278.9 ± 34.8	32	1.5
	30	104.7 ± 16.5	24	250.0 ± 26.9	24	2.4
	60	136.7 ± 19.9	24	247.9 ± 39.5	32	1.8
	120	175.5 ± 25.0	24	240.9 ± 27.3	24	1.4
**16a**	15	272.5 ± 21.4	32	430.9 ± 27.9	32	1.6
	30	181.7 ± 44.3	8	471.7 ± 26.7	32	2.6
	60	152.6 ± 23.5	24	455.8 ± 21.7	24	3.0
	120	140.9 ± 25.1	16	404.4 ± 24.0	24	2.9
**22a**	15	223.5 ± 20.9	24	341.0 ± 24.8	32	1.5
	30	228.3 ± 14.7	16	310.2 ± 26.2	32	1.4
	60	274.2 ± 10.9	8	433.2 ± 26.5	32	1.6
	120	448.1 ± 21.4	24	455.5 ± 29.7	32	1.0
**Valproate**	15	189.0 ± 17.3	24	363.3 ± 14.2	24	1.9
	30	216.9 ± 9.4	16	372.9 ± 16.9	16	1.7
	60	218.4 ± 18.9	24	417.3 ± 9.5	16	1.9
	120	246.6 ± 21.6	24	512.3 ± 20.2	32	2.1

Based on the data presented in Table 1 and Table 2, it may be assumed that the anticonvulsant activity of 1,2,4-triazole derivatives depends on the structure of substituents at positions N2, N4 and C5 of the triazole ring. In the group of monosubstituted derivatives of 4-aryl-5-(3-chlorophenyl)-2,4-dihydro-3*H*-1,2,4-triazole-3-thione (**1a**–**11a**), the most advantageous position for the substituent seemed to be the *para* position. Simultaneously, the electronic nature of the substituents was much less important as regards the effect on the anticonvulsant potency (since derivatives with both electron-withdrawing and electron-donating substituents were active). Moreover, the ED_50_ values for *para* substituted derivatives (**3a**, **4a**, **6a**, **7a**) allow the observation of the negative correlation between the anticonvulsant activity and the size of halogen substituent. Following this correlation, the 4-fluorophenyl derivative (**3a**) exhibited the most potent activity, whereas the 4-iodophenyl derivative (**7a**) was inactive. This may suggest that a large iodine atom constitutes a steric hindrance, which prevents the 4-halogenophenyl fragment from binding to appropriate molecular targets. Comparison of the activity of 2-fluorophenyl (**1a** *vs.* **12a**) and 2-bromophenyl (**5a** *vs.* **15a**) derivatives is more evidence confirming that the probability of obtaining compounds exerting anticonvulsant activity is much higher when at least one of the phenyl rings attached to the 1,2,4-triazole nucleus has a substituent at *para* position. Exchanging 3-chlorophenyl moiety (**1a**, **5a**) for a 4-chlorophenyl group (**12a**, **15a**) resulted in the emergence of anticonvulsant activity in the cases of 2-substituted derivatives.

In order to study the effect of the presence of a substituent at position N2, four active 1,2,4-triazoles (**6a**, **10a**, **15a**, **16a**) were transformed into their appropriate N2-aminomethyl derivatives (**19a**–**30a**). Among the compounds obtained this way, only compound **22a** exhibited a weak anticonvulsant activity, much less potent than that of its predecessor (**10a**). The remaining N2-aminomethyl derivatives were inactive. According to Waterbeemd and co-workers, the essential factors affecting the drug delivery across the blood-brain barrier (BBB) are molecular weight (M_W_) and polar surface area (PSA) [20]. Based on a study of marketed central nervous system (CNS)- and non-CNS drugs, it was established that the transport of a drug to the brain is possible when (T)PSA < 90Å, while M_W_ < 450. Among the Mannich bases (**19a**–**30a**), only compound **22a** met these requirements (Table 3). Therefore we speculate that the most probable cause of the loss of anticonvulsant activity, once the hydrogen atom is substituted with an aminomethyl moiety, is the loss of the ability of such compounds to penetrate across the blood brain barrier (BBB). Apart from the BBB, another important biological barrier the permeability of which (or lack thereof) affects drug efficiency, is the intestinal epithelium, which modulates drug absorption after oral administration. Most antiepileptic drugs are administered orally, with the exception of drugs interrupting *status epilepticus*. Therefore, newly synthesized compounds with antiepileptic potential should be efficiently absorbed from the digestive system. It has been demonstrated experimentally that intestinal absorption of drugs is significantly correlated with the (T)PSA parameter. Palm *et al*., have proven, based on Caco-2 cell studies, that the drugs with a (T)PSA below 60 are completely absorbed in the intestine [21]. In the cases of active compounds (**3a**, **4a**, **6a**, **10a**, **12a**, **13a**, **15a**, **16a**, and **22a**), the PSA values ranged from 33.62 to 35.23 Å^2^ and the level of intestinal absorption according to the algorithm described by Zhao *et al*., [22] was in the range of 96.8%–97.4% (Table 3). Using these calculations as a theoretical model it is highly likely that 1,2,4-triazole-based compounds could be considered as promising drug candidates, although further experiments should confirm their applicability in clinical settings.

**Table 3:** Physicochemical parameters of the studied 1,2,4-triazole-3-thiones.

Compounds	M_W_	logP	TPSA	ABS (%)
**1a**	305.765	3.817	33.617	97.40
**2a**	305.765	3.841	33.617	97.40
**3a**	305.765	3.654	33.617	97.40
**4a**	322.22	4.168	33.617	97.40
**5a**	366.671	4.462	33.617	97.40
**6a**	366.671	4.299	33.617	97.40
**7a**	413.671	4.573	33.617	97.40
**8a**	301.802	4.102	33.617	97.40
**9a**	301.802	4.126	33.617	97.40
**10a**	301.802	3.938	33.617	97.40
**11a**	329.812	3.388	50.688	91.51
**12a**	305.765	3.841	33.617	97.40
**13a**	323.755	3.981	33.617	97.40
**14a**	356.665	5.009	33.617	97.40
**15a**	366.671	4.486	33.617	97.40
**16a**	366.671	4.32	33.617	97.40
**17a**	333.869	3.947	33.617	97.40
**18a**	358.898	4.368	36.855	96.28
**19a**	465.804	4.23	35.232	96.84
**20a**	540.918	5.973	29.236	98.91
**21a**	558.908	6.136	29.236	98.91
**22a**	400.935	3.869	35.232	96.84
**23a**	476.049	5.612	29.236	98.91
**24a**	494.039	5.776	29.236	98.91
**25a**	465.804	4.417	35.232	96.84
**26a**	540.918	6.16	29.236	98.91
**27a**	558.908	6.324	29.236	98.91
**28a**	465.804	4.254	35.232	96.84
**29a**	540.918	5.997	29.236	98.91
**30a**	558.908	6.16	29.236	98.91

ABS (%)—absorption after oral administration.

#### 2.2.2. Radioligand Binding Assay

Epileptic seizures are caused by disruption of the balance between excitatory and inhibitory systems in the central nervous system. The most important neurotransmitter inhibiting neuronal processes is γ-aminobutyric acid (GABA). Among three types of GABA receptors (*i.e*., GABA_A_, GABA_B_, GABA_C_), the main role in preventing seizures is played by the GABA_A_ receptors [23,24]. Antiepileptic drugs acting directly on GABA_A_ receptors, enhancing the synthesis or inhibiting the metabolism of GABA, as well as blocking the reuptake of GABA from the synaptic cleft, cause an increase in the GABA-ergic neurotransmission [25]. Our studies conducted to date [12,13,26,27], involving the design and synthesis of 1,2,4-triazole derivatives endowed with anticonvulsant activity, have led to promising results, which in turn has encouraged us to attempt the elucidation of their mechanism of action. A representative group of the synthesized 1,2,4-triazole-based compounds (including active and inactive ones) was tested with the aim to establish whether the anticonvulsant activity of these derivatives was a result of their interactions with GABA_A_ receptor complex and/or their affinity to benzodiazepine (BDZ) binding sites. The degree of specific binding of the compounds to GABA_A_ receptors and BDZ-binding sites was low and ranged from 2% to 24% (Table 4). This forced us to exclude the possibility of a direct involvement of GABA_A_ receptors in the occurrence of the anticonvulsant effect. Similarly, the possibility of allosteric modulation of the function of this receptor via the interactions of the investigated 1,2,4-triazole derivatives with BDZ-binding sites should be excluded.

Activation of the GABA_A_ receptor may be due to binding of various chemical substances to the allosteric sites on the receptor surface [28]. Barbiturates and benzodiazepines are the best-known allosteric modulators of GABA_A_ receptors. However, a similar mechanism of action has also been described for loreclezole – a second-generation antiepileptic drug. Yet, the binding sites for this drug are distinct from those of the barbiturates and benzodiazepines. Loreclezole enhances GABA_A_-mediated chlorine currents via specific modulatory sites present on the β_2_ and β_3_ subunits of the GABA_A_ receptor [29]. Moreover, Wingrove and co-workers put forward a hypothesis that the activity of loreclezole is dependent on the interaction between its 1,2,4-triazole moiety and the amide group of asparagine (Asn-289), which is located on the β_2_ subunit of the GABA_A_ receptor [11]. This fact prompted us to check, whether the obtained 1,2,4-triazole derivatives may act via a mechanism analogous to that of loreclezole. For this study, two derivatives (**6a** and **10a**) were selected, as they are known to significantly elevate the seizure threshold and enhance the protective activity of classic antiepileptic drugs [12,13]. In the radioligand binding assay, the affinity of GABA administered alone or in combination with 1,2,4-triazole derivatives (**6a**, **10a**), was compared. The IC_50_ and K_i_ values for GABA administered alone were 406.5 ± 42.0 nM and 96.6 ± 9.9 nM, respectively (Table 5). The addition of **6a** and **10a** did not lead to an increase in the affinity of GABA to GABA_A_ receptors. The obtained results permit us to conclude that despite the structural similarity of loreclezole and compounds (**1a**–**30a**), their anticonvulsant activities are achieved via a completely different molecular mechanism.

**Table 4:** Affinity of the selected 1,2,4-triazole derivatives to GABA_A_ receptors and benzodiazepine (BDZ)-binding sites.

Compounds (Concentration 1 × 10^−6^ M)	Percent of Specific Binding (%)	
	GABA_A_	BDZ
**1a**	8	14
**5a**	2	12
**6a**	17	20
**10a**	2	9
**12a**	13	18
**15a**	8	21
**16a**	17	24
**17a**	21	6
**22a**	6	12
**23a**	23	7
**24a**	21	13
**GABA**	82	-
**Diazepam**	-	83
**Zolpidem**	-	73

**Table 5:** Effect of **6a** and **10a** on affinity of γ-aminobutyric acid (GABA) to GABA_A_ receptor.

Treatment	K_i_ ± S.E. [nM]	IC_50_ ± S.E. [nM]
GABA	96.6 ± 9.9	406.5 ± 42.0
GABA + **6a** (1 × 10^−5^ M)	99.7 ± 11.6	370.3 ± 42.7
GABA + **6a** (1 × 10^−6^ M)	108.0 ± 11.2	469.0 ± 55.9
GABA + **6a** (1 × 10^−7^ M)	155.0 ± 16.5	496.0 ± 57.0
GABA + **10a** (1 × 10^−5^ M)	112.3 ± 8.5	474.0 ± 36.2
GABA + **10a** (1 × 10^−6^ M)	85.5 ± 1.5	358.5 ± 6.5
GABA + **10a** (1 × 10^−7^ M)	107.0 ± 5.0	452.0 ± 22.0

## 3. Experimental Section

### 3.1. Chemistry

All reagents were purchased from Alfa-Aesar and Sigma-Aldrich and used without further purification. Melting points were determined by using Fischer-Johns apparatus (Sanyo, Japan) and are uncorrected. The ^1^H-NMR spectra were recorded on a Bruker Avance instrument using DMSO-*d_6_* or CDCl_3_ as solvents and tetramethylsilane (TMS) as an internal standard. Chemical shifts are expressed as *δ* (ppm). The infrared (IR) spectra were recorded in KBr using a Perkin-Elmer 1725X Fourier transform infrared (FTIR) spectrometer. The purity of the compounds was checked by thin-layer chromatography (TLC) on plates precoated with silica gel Si 60 F_254_, produced by Merck Co. (Darmstadt, Germany). The spots were detected by exposure to UV-lamp at λ = 254 nm. Elemental analyses were performed on AMZ 851 CHX analyzer and the results were within ±0.4% of the theoretical value. Molecular properties (*i.e.*, M_W_, TPSA, and logP) of the compounds were calculated using Molinspiration online tool [30].

#### 3.1.1. General Procedure for the Synthesis of Thiosemicarbazide Derivatives (**1**–**18**)

A solution of 0.01 mol of 3-chlorobenzhydrazide or 4-chlorobenzhydrazide and equimolar amount of appropriate aryl isothiocyanate in 25 mL of anhydrous ethanol (EtOH) was heated under reflux for 5 min. Next, the solution was cooled and the solid formed was filtered off, washed with diethyl ether and crystallized from EtOH. Information on already known compounds may be retrieved in the Chemical Abstract Service database (CAS numbers are given below).

*1-(3-Chlorobenzoyl)-4-(2-fluorophenyl)thiosemicarbazide* (**1**): Yield: 88%. CAS Registry Number: 894226-73-4.

*1-(3-Chlorobenzoyl)-4-(3-fluorophenyl)thiosemicarbazide* (**2**): Yield: 90%. CAS Registry Number: 894226-67-6.

*1-(3-Chlorobenzoyl)-4-(4-fluorophenyl)thiosemicarbazide* (**3**): Yield: 85%. CAS Registry Number: 316151-86-7.

*1-(3-Chlorobenzoyl)-4-(4-chlorophenyl)thiosemicarbazide* (**4**): Yield: 86%. CAS Registry Number: 351877-21-9.

*4-(2-Bromophenyl)-1-(3-chlorobenzoyl)thiosemicarbazide* (**5**): Yield: 86%, CAS Registry Number: 1263379-27-6.

*4-(4-Bromophenyl)-1-(3-chlorobenzoyl)thiosemicarbazide* (**6**): Yield: 85%. CAS Registry Number: 351877-22-0.

*1-(3-Chlorobenzoyl)-4-(4-iodophenyl)thiosemicarbazide* (**7**): Yield: 96%, CAS Registry Number: 1263379-28-7.

*1-(3-Chlorobenzoyl)-4-(2-methylphenyl)thiosemicarbazide* (**8**): Yield: 87%. CAS Registry Number: 891083-31-1.

*1-(3-Chlorobenzoyl)-4-(3-methylphenyl)thiosemicarbazide* (**9**): Yield: 86%. CAS Registry Number: 905231-64-3.

*1-(3-Chlorobenzoyl)-4-(4-methylphenyl)thiosemicarbazide* (**10**): Yield: 94%. CAS Registry Number: 891082-71-6

*4-(4-Acetylphenyl)-1-(3-chlorobenzoyl)thiosemicarbazide* (**11**): Yield: 86%. CAS Registry Number: 891560-05-7

*1-(4-Chlorobenzoyl)-4-(2-fluorophenyl)thiosemicarbazide* (**12**): Yield: 93%. CAS Registry Number: 894226-79-0.

*1-(4-Chlorobenzoyl)-4-(2,4-difluorophenyl)thiosemicarbazide* (**13**): Yield: 85%. CAS Registry Number: 891552-00-4.

*1-(4-Chlorobenzoyl)-4-(2,4-dichlorophenyl)thiosemicarbazide* (**14**): Yield: 90%. CAS Registry Number: 891643-33-7.

*4-(2-Bromophenyl)-1-(4-chlorobenzoyl)thiosemicarbazide* (**15**): Yield: 94%, m.p. 174–176 °C, ^1^H-NMR (300 MHz, DMSO-*d*_6_): 7.16–7.23 (m, 2H, Ar-H), 7.40–7.57 (m, 3H, Ar-H), 7.68–7.81 (m, 3H, Ar-H), 9.66 (s, 1H, 1NH, D_2_O exchangeable), 9.80 (s, 1H, 1NH, D_2_O exchangeable), 10.65 (s, 1H, NH, D_2_O exchangeable). IR (KBr, cm^−1^): 3367 (NH), 3110, 2991 (CH_arom._), 1652 (C=O), 1296 (C=S). EI-MS (*m/z*): 383 [M^+^]. Anal. Calc. for C_14_H_11_BrClN_3_OS (%): C 43.71, H 2.88, N 10.92. Found: C 43.65, H 2.72, N 11.05.

*4-(4-Bromophenyl)-1-(4-chlorobenzoyl)thiosemicarbazide* (**16**): Yield: 88%. CAS Registry Number: 356576-31-3.

*1-(4-Chlorobenzoyl)-4-[4-(methylsulfanyl)phenyl]thiosemicarbazide* (**17**): Yield: 96%, m.p. 180–182 °C, ^1^H-NMR (300 MHz, DMSO-d_6_): 2.26 (s, 3H, SCH_3_), 7.19 (d, 2H, Ar-H, *J* = 8.56 Hz), 7.30 (d, 2H, Ar-H, *J* = 8.55 Hz), 7.38–7.68 (m, 4H, Ar-H), 9.86 (s, 2H, 2NH, D_2_O exchangeable), 10.74 (s, 1H, NH, D_2_O exchangeable). IR (KBr, cm^−1^): 3410 (NH), 3076 (CH_arom_), 2930 (CH_aliph._), 1661 (C=O), 1311 (C=S). EI-MS (*m/z*): 351 [M^+^]. Anal. Calc. for C_15_H_14_ClN_3_OS_2_ (%): C 51.20, H 4.01, N 11.94. Found: C 51.03, H 4.11, N 11.98.

*1-(4-Chlorobenzoyl)-4-[4-(diethylamino)phenyl]thiosemicarbazide* (**18**): Yield: 87%, m.p. 158–159 °C, ^1^H-NMR (300 MHz, DMSO-*d*_6_): 1.08 (t, 6H, 2CH_3_, *J* = 6.80 Hz), 3.10 (q, 4H, 2CH_2_, *J* = 6.80 Hz), 6.94 (d, 2H, Ar-H, *J* = 8.65 Hz), 7.10 (d, 2H, Ar-H, *J* = 8.65 Hz), 7.45–7.81 (m, 4H, Ar-H), 9.56, 9.68, 10.54 (3s, 3H, 3NH, D_2_O exchangeable). IR (KBr, cm^−1^): 3386, 3350 (NH), 3064 (CH_arom_), 2951, 2863 (CH_aliph_), 1658 (C=O), 1304 (C=S). Anal. Calc. for C_18_H_21_ClN_4_OS (%): C 57.36, H 5.62, N 14.87. Found: C 57.42, H 5.79, N 14.76.

#### 3.1.2. General Procedure for the Synthesis of 1,2,4-triazole-3-thione Derivatives (**1a**–**18a**)

Suitable 1,4-disubstituted thiosemicarbazides (**1**–**18**) (0.01 mol) were dissolved in 2% solution of sodium hydroxide and the resulting solution was refluxed for 2 h. After cooling, the mixture was neutralized with 3M hydrochloric acid. The precipitate formed was filtered and washed with distilled water. The compounds were crystallized from EtOH.

*5-(3-Chlorophenyl)-4-(2-fluorophenyl)-2,4-dihydro-3H-1,2,4-triazole-3-thione* (**1a**): Yield: 81%, m.p. 170–171 °C, ^1^H-NMR (300 MHz, DMSO-*d*_6_): 7.18–7.77 (m, 8H, Ar-H), 14.17 (s, 1H, NH, D_2_O exchangeable). IR (KBr, cm^−1^): 3453 (NH), 3012 (CH_arom_), 1485 (C=N), 1321 (C=S). EI-MS (*m/z*): 305 [M^+^]. Anal. Calc. for C_14_H_9_ClFN_3_S (%): C 54.99, H 2.97, N 13.74. Found: C 55.06, H 2.76, N 13.88.

*5-(3-Chlorophenyl)-4-(3-fluorophenyl)-2,4-dihydro-3H-1,2,4-triazole-3-thione* (**2a**): Yield: 83%, m.p. 221–222 °C, ^1^H-NMR (300 MHz, DMSO-*d*_6_): 7.17–7.62 (m, 8H, Ar-H), 14.27 (s, 1H, NH, D_2_O exchangeable). IR (KBr, cm^−1^): 3461 (NH), 3002 (CH_arom._), 1483 (C=N), 1320 (C=S). EI-MS (*m/z*): 305 [M^+^]. Anal. Calc. for C_14_H_9_ClFN_3_S (%): C 54.99, H 2.97, N 13.74. Found: C 55.08, H 2.81, N 13.85.

*5-(3-Chlorophenyl)-4-(4-fluorophenyl)-2,4-dihydro-3H-1,2,4-triazole-3-thione* (**3a**): Yield: 90%. CAS Registry Number: 720667-75-4.

*4-(4-Chlorophenyl)-5-(3-chlorophenyl)-2,4-dihydro-3H-1,2,4-triazole-3-thione* (**4a**): Yield: 82%. CAS Registry Number: 879074-80-3.

*4-(2-Bromophenyl)-5-(3-chlorophenyl)-2,4-dihydro-3H-1,2,4-triazole-3-thione* (**5a**): Yield: 85%. CAS Registry Number: 1263379-33-4.

*4-(4-Bromophenyl)-5-(3-chlorophenyl)-2,4-dihydro-3H-1,2,4-triazole-3-thione* (**6a**): Yield: 95%. CAS Registry Number: 1263379-32-3.

*5-(3-Chlorophenyl)-4-(4-iodophenyl)-2,4-dihydro-3H-1,2,4-triazole-3-thione* (**7a**): Yield: 87%. CAS Registry Number: 1263379-34-5.

*5-(3-Chlorophenyl)-4-(2-methylphenyl)-2,4-dihydro-3H-1,2,4-triazole-3-thione* (**8a**): Yield: 87%, m.p. 186–188 °C, ^1^H-NMR (300 MHz, DMSO-*d*_6_): 2.13 (s, 3H, CH_3_), 7.12–7.86 (m, 8H, Ar-H), 14.17 (s, 1H, NH, D_2_O exchangeable). IR (KBr, cm^−1^): 3387 (NH), 3046 (CH_arom._), 2931 (CH_aliph_), 1494 (C=N), 1332 (C=S). EI-MS (*m/z*): 301 [M^+^]. Anal. Calc. for C_15_H_12_ClN_3_S (%):C 59.70, H 4.01, N 13.92. Found: C 59.79, H 3.87, N 14.03.

*5-(3-Chlorophenyl)-4-(3-methylphenyl)-2,4-dihydro-3H-1,2,4-triazole-3-thione* (**9a**): Yield: 83%, m.p. 200–201 °C, ^1^H-NMR (300 MHz, DMSO-*d*_6_): 2.32 (s, 3H, CH_3_), 7.10–7.53 (m, 8H, Ar-H), 14.09 (s, 1H, NH, D_2_O exchangeable). IR (KBr, cm^−1^): 3415 (NH), 3068 (CH_arom._), 2864 (CH_aliph._), 1476 (C=N), 1322 (C=S). EI-MS (*m/z*): 301 [M^+^]. Anal. Calc. for C_15_H_12_ClN_3_S (%):C 59.70, H 4.01, N 13.92. Found: C 59.81, H 4.09, N 14.13.

*5-(3-Chlorophenyl)-4-(4-methylphenyl)-2,4-dihydro-3H-1,2,4-triazole-3-thione* (**10a**): Yield: 80%. CAS Registry Number: 893725-08-1.

*4-(4-Acetylphenyl)-5-(3-chlorophenyl)-2,4-dihydro-3H-1,2,4-triazole-3-thione* (**11a**): Yield: 84%, m.p. 218–220 °C, ^1^H-NMR (300 MHz, DMSO-*d*_6_): 2.64 (s, 3H, CH_3_), 7.21–8.14 (m, 8H, Ar-H), 14.27 (s, 1H, NH, D_2_O exchangeable). IR (KBr, cm^−1^): 3421 (NH), 3041 (CH_arom._), 2924 (CH_alif._), 1666 (C=O), 1490 (C=N), 1328 (C=S). Anal. Calc. for C_16_H_12_ClN_3_OS (%): C 58.27, H 3.67, N 12.74. Found: C 58.14, H 3.58, N 12.67.

*5-(4-Chlorophenyl)-4-(2-fluorophenyl)-2,4-dihydro-3H-1,2,4-triazole-3-thione* (**12a**): Yield: 86%, m.p. 240–242 °C, ^1^H-NMR (300 MHz, DMSO-*d*_6_): 7.40 (d, 2H, Ar-H, *J* = 8.70 Hz), 7.52 (d, 2H, Ar-H, *J* = 8.69 Hz), 7.58–7.86 (m, 4H, Ar-H), 14.32 (s, 1H, NH, D_2_O exchangeable). IR (KBr, cm^−1^): 3387 (NH), 3034 (CH_arom._), 1489 (C=N), 1328 (C=S). Anal. Calc. for C_14_H_9_ClFN_3_S (%): C 54.99, H 2.97, N 13.74. Found: C 55.10, H 3.00, N 13.61.

*5-(4-Chlorophenyl)-4-(2,4-difluorophenyl)-2,4-dihydro-3H-1,2,4-triazole-3-thione* (**13a**): Yield: 86%, m.p. 196–198 °C, ^1^H-NMR (300 MHz, DMSO-*d*_6_): 7.24 (d, 2H, Ar-H, *J* = 8.63 Hz), 7.42 (d, 2H, Ar-H, *J* = 8.61 Hz), 7.58–7.81 (m, 3H, Ar-H), 14.29 (s, 1H, NH, D_2_O exchangeable). IR (KBr, cm^−1^): 3420 (NH), 3100 (CH_arom._), 1483 (C=N), 1336 (C=S). EI-MS (*m/z*): 323 [M^+^]. Anal. Calc. for C_14_H_8_ClF_2_N_3_S (%): C 51.94, H 2.49, N 12.98. Found: C 52.13, H 2.50, N 12.81.

*5-(4-Chlorophenyl)-4-(2,4-dichlorophenyl)-2,4-dihydro-3H-1,2,4-triazole-3-thione* (**14a**): Yield: 88%, m.p. 238–240 °C, ^1^H-NMR (300 MHz, DMSO-*d*_6_): 7.39 (d, 2H, Ar-H, *J* = 8.65 Hz), 7.55 (d, 2H, Ar-H, *J* = 8.66 Hz), 7.70–7.95 (m, 3H, Ar-H), 14.39 (s, 1H, NH, D_2_O exchangeable). IR (KBr, cm^−1^): 3387 (NH), 3049 (CH_arom._), 1490 (C=N), 1318 (C=S). Anal. Calc. for C_14_H_8_Cl_3_N_3_S (%): C 47.15, H 2.26, N 11.78. Found: C 47.13, H 2.30, N 11.66.

*4-(2-Bromophenyl)-5-(4-chlorophenyl)-2,4-dihydro-3H-1,2,4-triazole-3-thione* (**15a**): Yield: 90%, m.p. 250–252 °C, ^1^H-NMR (300 MHz, DMSO-*d*_6_): 7.36 (d, 2H, Ar-H, *J* = 8.61 Hz), 7.50 (d, 2H, Ar-H, *J* = 8.61 Hz), 7.67–7.91 (m, 4H, Ar-H), 14.30 (s, 1H, NH, D_2_O exchangeable). IR (KBr, cm^−1^): 3401 (NH), 3105 (CH_arom_), 1496 (C=N), 1342 (C=S). EI-MS (*m/z*): 365 [M^+^]. Anal. Calc. for C_14_H_9_BrClN_3_S (%): C 45.86, H 2.47, N 11.46. Found: C 45.65, H 2.53, N 11.60.

*4-(4-Bromophenyl)-5-(4-chlorophenyl)-2,4-dihydro-3H-1,2,4-triazole-3-thione* (**16a**): Yield: 84%, CAS Registry Number: 537017-82-6.

*5-(4-Chlorophenyl)-4-[4-(methylsulfanyl)phenyl]-2,4-dihydro-3H-1,2,4-triazole-3-thione* (**17a**): Yield: 88%, m.p. 214–216 °C, ^1^H-NMR (300 MHz, DMSO-*d*_6_): 2.42 (s, 3H, CH_3_), 7.23–7.85 (m, 8H, Ar-H), 14.29 (s, 1H, NH, D_2_O exchangeable). IR (KBr, cm^−1^): 3412 (NH), 3075 (CH_arom_), 2886 (CH_aliph._), 1568 (C=N), 1320 (C=S). EI-MS (*m/z*): 333 [M^+^]. Anal. Calc. for C_15_H_12_ClN_3_S_2_ (%): C 53.96, H 3.62, N 12.59. Found: C 54.10, H 3.54, N 13.02.

*5-(4-Chlorophenyl)-4-[4-(diethylamino)phenyl]-2,4-dihydro-3H-1,2,4-triazole-3-thione* (**18a**): Yield: 80%, m.p. 264–266 °C, ^1^H-NMR (300 MHz, DMSO-*d*_6_): 1.10 (t, 6H, 2 × CH_3_, *J* = 7.08 Hz), 3.34 (q, 4H, 2CH_2_, *J* = 7.10 Hz), 7.10 (d, 2H, Ar-H, *J* = 8.80 Hz), 7.21 (d, 2H, Ar-H, *J* = 8.80 Hz), 7.28–7.61 (m, 4H, Ar-H), 14.23 (s, 1H, NH, D_2_O exchangeable). IR (KBr, cm^−1^): 3422 (NH), 3041 (CH_arom._), 2926, 2864 (CH_aliph._), 1559 (C=N), 1330 (C=S). Anal. Calc. for C_18_H_19_ClN_4_S (%): C 60.24, H 5.34, N 15.61. Found: C 60.31, H 5.48, N 15.60.

#### 3.1.3. General Procedure for the Synthesis of Mannich Bases (**19a**–**30a**)

Ten mmol of the 1,2,4-triazole derivative (**6a**, **10a**, **15a**, **16a**) was dissolved (with heating) in 20 mL of anhydrous ethanol and then equimolar amounts of appropriate secondary amine (morpholine, 1-phenylpiperazine, 1-[4-fluorophenyl]piperazine) and formaldehyde solution (37%) were added. The obtained mixture was stirred at room temperature for 1 h. Next, 5 mL of distilled water was added, the precipitate was filtered off, washed with distilled water, and crystallized from ethanol. Spectral and physicochemical data for compounds **19a**–**24a**, and **28a** are available in our earlier articles [31,32].

*4-(4-Bromophenyl)-5-(3-chlorophenyl)-2-(morpholin-4-ylmethyl)-2,4-dihydro-3H-1,2, 4-triazole-3-thione* (**19a**): Yield: 75%. CAS Registry Number: 1349172-90-2.

*4-(4-Bromophenyl)-5-(3-chlorophenyl)-2-[(4-phenylpiperazin-1-yl)methyl]-2,4-dihydro-3H-1,2, 4-triazole-3-thione* (**20a**): Yield: 78%. CAS Registry Number: 1349172-92-4.

*4-(4-Bromophenyl)-5-(3-chlorophenyl)-2-{[4-(4-fluorophenyl)piperazin-1-yl]methyl}-2, 4-dihydro-3H-1,2,4-triazole-3-thione* (**21a**): Yield: 82%. CAS Registry Number: 1349172-94-6.

*5-(3-Chlorophenyl)-4-(4-methylphenyl)-2-(morpholin-4-ylmethyl)-2,4-dihydro-3H-1,2, 4-triazole-3-thione* (**22a**): Yield: 72%. CAS Registry Number: 1349172-89-9.

*5-(3-Chlorophenyl)-4-(4-methylphenyl)-2-[(4-phenylpiperazin-1-yl)methyl]-2,4-dihydro-3H-1,2, 4-triazole-3-thione* (**23a**): Yield: 79%. CAS Registry Number: 1349172-91-3.

*5-(3-Chlorophenyl)-2-{[4-(4-fluorophenyl)piperazin-1-yl]methyl}-4-(4-methylphenyl)-2,4-dihydro-3H-1,2,4-triazole-3-thione* (**24a**): Yield: 83%. CAS Registry Number: 1349172-93-5.

*4-(2-Bromophenyl)-5-(4-chlorophenyl)-2-(morpholin-4-ylmethyl)-2,4-dihydro-3H-1,2,4-triazole-3-thione* (**25a**): Yield: 80%, m.p. 180–181 °C, ^1^H-NMR (300 MHz, CDCl_3_): 2.93 (t, 4H, morpholine, *J* = 4.70 Hz), 3.75 (t, 4H, morpholine, *J* = 4.70 Hz), 5.25 (s, 2H, CH_2_), 7.20 (d, 2H, Ar-H, *J* = 8.60 Hz), 7.26–7.34 (m, 4H, Ar-H), 7.65 (d, 2H, Ar-H, *J* = 8.60 Hz). ^13^C-NMR (75 MHz, CDCl_3_): 50.86, 66.90, 70.05, 123.49, 124.20, 129.23, 129.43, 129.53, 129.77, 129.89, 133.14, 133.85, 137.21, 148.21, 170.61. EI-MS (*m/z*): 464 [M^+^]. Anal. Calc. for C_19_H_18_BrClN_4_OS (%): C 48.99, H 3.90, N 12.03. Found: C 49.12, H 3.78, N 12.00.

*4-(2-Bromophenyl)-5-(4-chlorophenyl)-2-[(4-phenylpiperazin-1-yl)methyl]-2,4-dihydro-3H-1,2, 4-triazole-3-thione* (**26a**): Yield: 73%, m.p. 140–142 °C, ^1^H-NMR (600 MHz, CDCl_3_): 3.09–3.20 (m, 4H, piperazine), 3.27 (t, 4H, piperazine, *J* = 4.9 Hz), 5.31 (d, 1H, CH_2_, *J* = 13.2 Hz), 5.48 (d, 1H, CH_2_, *J* = 13.2 Hz), 6.89 (t, 1H, Ar-H, *J* = 7.2 Hz), 6.97 (d, 2H, Ar-H, *J* = 7.4 Hz), 7.27–7.32 (m, 5H, Ar-H), 7.34 (dd, 2H, Ar-H, *J* = 1.9 Hz, 5.9 Hz), 7.42–7.48 (m, 2H, Ar-H), 7.55 (td, 1H, Ar-H, *J* = 1.2 Hz, 7.6 Hz), 7.75 (dd, 1H, Ar-H, *J* = 1.2 Hz, 7.9 Hz). ^13^C NMR (150 MHz, CDCl_3_): 49.48, 50.40, 69.63, 116.41, 123.35, 123.87, 129.07, 129.16, 129.19, 130.92, 131.91, 134.20, 134.40, 137.12, 148.20, 170.43. IR (KBr, cm^−1^): 3084, 3006 (CH_arom_), 2892, 2784 (CH_aliph_), 1495 (C=N), 1314 (C=S). Anal. Calc. for C_25_H_23_BrClN_5_S (%):C 55.51, H 4.29, N 12.95. Found: C 55.57, H 4.12, N 12.79.

*4-(2-Bromophenyl)-5-(4-chlorophenyl)-2-{[4-(4-fluorophenyl)piperazin-1-yl]methyl}-2, 4-dihydro-3H-1,2,4-triazole-3-thione* (**27a**): Yield: 82%, m.p. 168–170 °C, ^1^H-NMR (600 MHz, CDCl_3_): 3.09–3.25 (m, 8H, piperazine), 5.30 (d, 1H, CH_2_, *J* = 13.4 Hz), 5.47 (d, 1H, CH_2_, *J* = 13.4 Hz), 6.89-6.96 (m, 2H, Ar-H), 6.99 (t, 2H, Ar-H, *J* = 8.1 Hz), 7.27–7.36 (m, 4H, Ar-H), 7.42–7.47 (m, 2H, Ar-H), 7.55 (td, 1H, Ar-H, *J* = 1.3 Hz, 7.6 Hz), 7.75 (dd, 1H, Ar-H, *J* = 1.3 Hz, 8.0 Hz). ^13^C NMR (150 MHz, CDCl_3_): 50.32, 50.54, 69.51, 115.57, 115.72, 123.34, 123.83, 129.06, 129.16, 130.90, 131.92, 134.20, 134.38, 137.16, 148.23, 170.44. IR (KBr, cm^−1^): 3130, 3085 (CH_arom._), 2956, 2879 (CH_aliph._), 1486 (C=N), 1317 (C=S). Anal. Calc. for C_25_H_22_BrClFN_5_S (%): C 53.73, H 3.97, N 12.53. Found: C 53.80, H 3.76, N 12.69.

*4-(4-Bromophenyl)-5-(4-chlorophenyl)-2-(morpholin-4-ylmethyl)-2,4-dihydro-3H-1,2,4-triazole-3-thione* (**28a**): Yield: 69%. CAS Registry Number: 1403137-49-4.

*4-(4-Bromophenyl)-5-(4-chlorophenyl)-2-[(4-phenylpiperazin-1-yl)methyl]-2,4-dihydro-3H-1,2,4-triazole-3-thione* (**29a**): Yield: 78%, m.p. 178–180 °C, ^1^H-NMR (CDCl_3_): 3.12–3.15 (m, 4H, piperazine), 3.25–3.28 (m, 4H, piperazine), 5.37 (s, 2H, CH_2_), 6.90 (t, 1H, Ar-H, *J* = 7.30 Hz), 6.96 (d, 2H, Ar-H, *J* = 8.20 Hz), 7.21 (d, 2H, Ar-H, *J* = 8.60 Hz), 7.25–7.39 (m, 6H, Ar-H), 7.69 (d, 2H, Ar-H, *J* = 8.60 Hz). ^13^C NMR (CDCl_3_): 49.50, 50.60, 69.89, 116.51, 120.16, 123.56, 124.23, 129.23, 129.28, 129.61, 129.95, 133.19, 133.92, 137.20, 148.20, 151.31, 170.60. EI-MS (*m/z*): 539 [M^+^]. Anal. Calc. for C_25_H_23_BrClN_5_S (%): C 55.51, H 4.29, N 12.95. Found: C 55.38, H 4.38, N 13.12.

*4-(4-Bromophenyl)-5-(4-chlorophenyl)-2-{[4-(4-fluorophenyl)piperazin-1-yl]methyl}-2, 4-dihydro-3H-1,2,4-triazole-3-thione* (**30a**): Yield: 80%, m.p. 156–158 °C, ^1^H-NMR (CDCl_3_): 3.07–3.20 (m, 8H, piperazine), 5.35 (s, 2H, CH_2_), 6.87–7.02 (m, 4H, Ar-H), 7.19 (d, 2H, Ar-H, *J* = 8.40 Hz), 7.24–7.32 (m, 4H, Ar-H), 7.66 (d, 2H, Ar-H, *J* = 8.40 Hz). ^13^C NMR (CDCl_3_): 50.50, 50.55, 69.78, 115.47, 115.77, 118.25, 118.35, 123.49, 124.24, 129.25, 129.54, 129.88, 133.18, 133.86, 137.23, 147.90, 148.20, 170.57. EI-MS (*m/z*): 557 [M^+^]. Anal. Calc. for C_25_H_22_BrClFN_5_S (%): C 53.73, H 3.97, N 12.53. Found: C 53.87, H 4.11, N 12.45.

### 3.2. Pharmacology

#### 3.2.1. General Information

Adult male Swiss mice (weighing 22–26 g) that were kept in colony cages with free access to food and tap water, housed under standardized housing conditions, were used. After seven days of adaptation to laboratory conditions, the animals were randomly assigned to experimental groups each comprised of eight mice. Each mouse was used only once. Procedures involving animals and their care were conducted in accordance with current European Community and Polish legislation on animal experimentation. The experimental protocols and procedures described in this manuscript were approved by the First Local Ethics Committee at the Medical University in Lublin and the Second Local Ethics Committee at the University of Life Sciences in Lublin and complied with the European Communities Council Directive of 24 November 1986 (86/609/EEC).

#### 3.2.2. Maximal Electroshock Seizure Test

The investigated compounds (**1a**–**30a**) were suspended in a 1% solution of Tween 80 in distilled water and administered intraperitoneally (*i.p.*) as a single injection, in a volume of 5 mL/kg body weight. Magnesium valproate, used as a reference drug, was directly dissolved in distilled water. Fresh drug solutions were prepared on each day of experimentation and administered at 15, 30, 60 and 120 min. before the initiation of maximal electroconvulsions. Electroconvulsions were produced by a current (0.2 s stimulus duration; 500 V, 50 Hz, fixed current intensity of 25 mA) delivered *via* ear-clip electrodes by a Rodent Shocker generator (constant-current stimulator Type 221, Hugo Sachs Elektronik, Freiburg, Germany). The criterion for the occurrence of seizure activity was the tonic hind limb extension. The animals were administered with a constant dose of 300 mg/kg of each of the examined compounds and were subjected to MES-induced seizures. The anticonvulsant activity of the active compounds administered *i.p.* at various pretreatment times was determined as their median effective doses (ED_50_ values in mg/kg) in the MES-induced seizure test in mice. The animals were administered with different doses of the tested compounds so as to obtain a variable percentage of protection against maximal electroconvulsions, allowing the construction of dose-response relationship lines for each examined compound administered *i.p*. at various pretreatment times, according to Litchfield and Wilcoxon [33]. Each ED_50_ value represents the dose of the studied compounds that is required to protect 50% of the animals tested against MES-induced seizures.

#### 3.2.3. Chimney Test

The chimney test of Boissier *et al*. [34] was used to quantify the acute adverse-effect potential of compounds **3a**, **4a**, **6a**, **10a**, **12a**, **13a**, **15a**, **16a**, **22a**, and valproate on motor performance in mice. In this test, the animals had to climb backwards up a plastic tube (3 cm inner diameter, 30 cm length), and motor performance impairment was indicated by the inability of the mice to climb backward up the transparent tube within 60 s. The acute adverse effects of the 1,2,4-triazole derivatives and valproate administered alone were expressed as their median toxic doses (TD_50_), representing the doses, at which the investigated compounds impaired motor coordination in 50% of the animals tested in the chimney test. To evaluate each TD_50_ value, at least four groups of animals (each group consisted of eight mice) injected with various doses of the appropriate compound were challenged with the chimney test. A dose-response relationship line was calculated on the basis of the percentage of mice showing motor deficits by means of the log-probit method according to Litchfield and Wilcoxon [33].

#### 3.2.4. Protective Index (PI)

The protective index for the investigated compounds was calculated by dividing a TD_50_ value, as determined in the chimney test, by the respective ED_50_ value, as determined in the MES test. The protective index is considered as an index of the margin of safety and tolerability between anticonvulsant doses and doses of the compounds exerting acute adverse effects e.g., sedation, motor coordination impairment, ataxia or other neurotoxic manifestations [35].

#### 3.2.5. Radioligand Binding Assay

Binding experiments were conducted in 96-well microplates in a total volume of 200 μL of appropriate buffer. The reaction mixture included 50 μL solution of test compound, 50 μL of radioligand, and 150 μL the tissue suspension. Specific assay conditions for each receptor are shown in the table below (Table 6). Rat cerebral cortex was used for each receptor. The radioactivity was measured in MicroBeta TriLux 1450-liquid scintillation counter (PerkinElmer, Waltham, MA, USA). Radioligand binding data were analyzed using iterative curve fitting routines (GraphPAD/Prism, Version 3.0, San Diego, CA, USA). K_i_ values were calculated from the Cheng and Prusoff equation [36]. The concentrations of analyzed compound GABA (γ-aminobutyric acid) ranged from 1 × 10^−11^ to 1 × 10^−3^ M. The percentage of specific binding was calculated for analyzed compounds in the screening test, which was determined in a concentration of 10^−6^ M.

**Table 6:** Radioligand binding assay conditions.

Receptor	Radioligand	Blank(nonspecific)	Buffer	Incubation Conditions
GABA_A_	[^3^H]muscimol	100 µM GABA	50 mM Tris-HCl pH 7.4	10 min, 0–4 °C
BDZ	[^3^H]flunitrazepam	10 µM diazepam	50 mM Tris-HCl pH 7.4	20 min, 0–4 °C

## 4. Conclusions

In conclusion, a series of 1,2,4-triazole-based compounds has been synthesized and their anticonvulsant and neurotoxic effects have been evaluated. Nine out of 30 tested derivatives exhibited various level of anticonvulsant activity. Median effective dose (ED_50_) for the most potent compound, *i.e*., 5-(3-chlorophenyl)-4-(4-fluorophenyl)-2,4-dihydro-3*H*-1,2,4‑triazole-3-thione, equaled 35.2 mg/kg. Based on the radioligand binding assay results, the hypothesis that the anticonvulsant activity of the mentioned compounds results from the direct or allosteric modulation of GABA_A_ receptors should be excluded.
